# The Impact of Monaural Beat Stimulation on Anxiety and Cognition

**DOI:** 10.3389/fnhum.2017.00251

**Published:** 2017-05-15

**Authors:** Leila Chaieb, Elke C. Wilpert, Christian Hoppe, Nikolai Axmacher, Juergen Fell

**Affiliations:** ^1^Department of Epileptology, University of BonnBonn, Germany; ^2^Department of Neuropsychology, Faculty of Psychology, Institute of Cognitive Neuroscience, University of BochumBochum, Germany

**Keywords:** auditory beat stimulation, monaural beats, mood states, anxiety, vigilance, long-term memory, working memory

## Abstract

Application of auditory beat stimulation has been speculated to provide a promising new tool with which to alleviate symptoms of anxiety and to enhance cognition. In spite of reportedly similar EEG effects of binaural and monaural beats, data on behavioral effects of monaural beats are still lacking. Therefore, we examined the impact of monaural beat stimulation on anxiety, mood and memory performance. We aimed to target states related to anxiety levels and general well-being, in addition to long-term and working memory processes, using monaural beats within the range of main cortical rhythms. Theta (6 Hz), alpha (10 Hz) and gamma (40 Hz) beat frequencies, as well as a control stimulus were applied to healthy participants for 5 min. After each stimulation period, participants were asked to evaluate their current mood state and to perform cognitive tasks examining long-term and working memory processes, in addition to a vigilance task. Monaural beat stimulation was found to reduce state anxiety. When evaluating responses for the individual beat frequencies, positive effects on state anxiety were observed for all monaural beat conditions compared to control stimulation. Our results indicate a role for monaural beat stimulation in modulating state anxiety and are in line with previous studies reporting anxiety-reducing effects of auditory beat stimulation.

## Introduction

Emerging studies have shown that auditory beat stimulation can affect mood states in terms of levels of anxiety (Le Scouarnec et al., 2001; Padmanabhan et al., 2005; Weiland et al., 2011) and well-being (Lane et al., 1998; Le Scouarnec et al., 2001; Wahbeh et al., 2007a). The application of auditory beats either monaurally (i.e., physical beats delivered to both ears) or binaurally (two different sine waves of neighboring frequencies delivered to each ear separately), may also be a promising new tool with which to target cognition in a reversible, non-invasive way. However, the majority of studies looking at the effects of auditory beats have been performed using binaural beat stimuli despite evidence suggesting that cortical responses to monaural beat stimuli are stronger than those to binaural beats (Schwarz and Taylor, 2005; Draganova et al., 2008).

**Figure 1:**
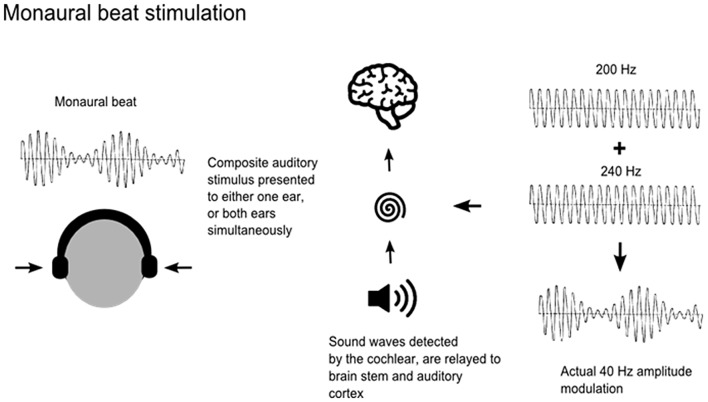
**Application of monaural beats**. Monaural beats can be applied by the superposition of amplitude modulated signals of nearby frequencies delivered either to one ear or to both ears. Carrier tones of 200 Hz and 240 Hz generating a 40 Hz beat are shown here as an example.

Auditory beats are amplitude-modulated signals, which can be generated by the superposition of two auditory sine waves with neighboring frequencies, in one of two ways. Monaural beat stimulation is achieved by applying the same amplitude-modulated signal to both ears simultaneously. This physical beat signal is modulated first in the cochlea and then relayed via brain stem neurons to the auditory cortex (Figure 1). As both ears receive the same beat wave, perception of the beat does not require an integration of information from the two ears (Pratt et al., 2009). In contrast, binaural beats occur when sine waves with neighboring frequencies are presented to each ear separately. For example, presentation of 40 Hz oscillations to the left ear and 45 Hz oscillations to the right ear results in the perception of an amplitude-modulated (“beat”) stimulus of 5 Hz. The binaural beat sensation is often described as being subjectively located “inside” the head and is understood to be modulated at the level of the brainstem in the superior olivary nuclei, whereas monaural stimuli are modulated at the level of the cochlea (Regan and Regan, 1988; Draganova et al., 2008). Previous studies investigating steady-state responses and the topographical distribution of cortical responses to monaural and binaural beats have reported maxima at frontocentral, mid-sagittal and temporal regions for both monaural and binaural beat stimuli (Karino et al., 2006; Draganova et al., 2008; Pratt et al., 2009). However, responses to monaural beat stimuli were more pronounced, at all beat frequencies applied (Schwarz and Taylor, 2005; Pratt et al., 2010). Nevertheless, previous studies examining the effects of auditory beat stimulation on anxiety, mood states and cognition have used binaural beats (Le Scouarnec et al., 2001; Padmanabhan et al., 2005; Wahbeh et al., 2007a,b).

Binaural beats were reported to affect anxiety scores in several studies. For instance, Padmanabhan et al. (2005) applied delta frequency binaural beat stimuli to patients suffering from pre-operative anxiety. STAI-S scores (assessed using the state-trait anxiety inventory, STAI) indicated a reduction in state-anxiety post stimulation. Daily application of binaural beats in the delta/theta range was also found to reduce STAI-S state-anxiety ratings in participants suffering from mild anxiety disorders (Le Scouarnec et al., 2001). Binaural beats at delta frequency (2.5 Hz) were reported to reduce trait-anxiety in healthy participants after 40 min application (Wahbeh et al., 2007a). To summarize, there is converging evidence for a reduction of anxiety by binaural beat stimulation.

Regarding the effects of binaural beats on mood and cognition, in a study in healthy participants using theta (7 Hz) frequency binaural beats again applied for 30 min an increase of the Profile Of Mood States (POMS) depression subscale scores and decreased verbal memory recall in the Rey Auditory Verbal Learning Test, compared to the control condition were observed (Wahbeh et al., 2007b). Other studies have reported enhancing effects on verbal memory for theta-frequency beats (Ortiz et al., 2008) and increased vigilance and less negative mood for beta-frequency compared to delta and theta frequency beats (Lane et al., 1998; Ortiz et al., 2008). Moreover, behavioral effects of alpha (10 Hz) and gamma (40 Hz) frequency binaural beats on creativity (Reedijk et al., 2013; alpha and gamma), cognitive flexibility (Hommel et al., 2016; gamma), as well as on attentional control (Reedijk et al., 2015; alpha and gamma) and attentional focusing have been described (Colzato et al., 2017; gamma). Interestingly, the effects on creativity and attentional control depended on an adjunct measure of individual striatal dopamine levels (spontaneous eye blink rates). Furthermore, increased response accuracies in a visuospatial working memory task due to beta (15 Hz) binaural beats have been recently reported (Beauchene et al., 2016). In summary, there is only sparse evidence, up to now, for effects of binaural beats on other putative applications than anxiety reduction. Moreover, the impact of monaural beat stimulation with regard to modulating mood states and cognition remains largely unknown.

It is important to note that studies investigating the effects of beat stimuli vary widely as to the stimulation duration applied. A recent study examining the effects of both binaural and monaural beat stimulation, using intracranial EEG data recorded from presurgical epilepsy patients, showed that short duration beat stimulation (5 s) altered measures of EEG power and phase synchronization (Becher et al., 2015). Five hertz binaural beats increased phase synchronization at temporo-lateral sites while 5 Hz monaural beats decreased mediotemporal phase synchronization (Becher et al., 2015). Another study applied monaural and binaural beats for a duration of 40–400 s and reported a phase shift in auditory steady-state responses from frontal to occipital sites at 40 Hz (Schwarz and Taylor, 2005). Earlier studies examining the effects of binaural beat stimulation on anxiety measures used longer stimulation durations of up to 30–60 mins daily (Le Scouarnec et al., 2001; Wahbeh et al., 2007a). We chose to apply monaural beats for 5 min during each test battery block so that cumulatively, participants would not exceed a total of 15 min of monaural beat stimulation to avoid possible side effects. The side effects of auditory beat stimulation, for example, the potential for fatiguing effects and headaches, have not yet been categorically investigated.

In the current study we investigated the effects of monaural beat stimulation on a number of neuropsychological measures. Although we aimed primarily to examine the effects of beat stimulation on state-anxiety, we also tested whether other interrelated neuropsychological factors could be influenced. To this end, we used an exploratory approach to investigate the impact of beat stimulation on mood states, vigilance, working and long-term memory. We implemented a counter-balanced within-subject study design to eliminate effects related to inter-individual variability. As EEG responses to monaural beats are topographically similar to and even stronger than those of binaural beats (Schwarz and Taylor, 2005; Pratt et al., 2009), we hypothesized that reductions of state-anxiety may be observed for monaural beat stimulation, similar or even more pronounced than those reported in the literature for binaural beat stimulation.This hypothesis is particularly related to the 10 Hz and 6 Hz stimulation conditions used in the present investigation, as previous anxiety-related studies applied binaural beats with frequencies at or below the alpha range. Since the reported effects of binaural beat stimulation on mood and memory are somewhat divergent, we had no clear directional hypotheses regarding the effects of monaural beat stimulation on these measures. However, we did speculate that 6 Hz monaural stimulation might reduce long-term memory performance, as we found decreased mediotemporal phase synchronization due to theta monaural beat stimulation in a previous study (Becher et al., 2015).

Beat stimuli were comprised of 6 Hz, 10 Hz and 40 Hz amplitude modulations, thus corresponding to dominant EEG frequencies in the theta, alpha and gamma band, in addition to a pure sine wave control condition. We chose these beat stimuli as previous studies have reported decreases in anxiety rating subscales using similar frequencies (theta and alpha; Le Scouarnec et al., 2001; Weiland et al., 2011), and because these frequencies (theta and gamma) have also been associated with long-term and working memory processes (e.g., Fell and Axmacher, 2011).

## Materials and Methods

### Participants

Twenty-five healthy subjects (12 male, age range: 24.4 ± SD: 2 years) were included in the study. Participants were contacted using information from a participant study database in a non-selective manner per email. The first 25 participants who replied and met inclusion criteria were recruited to join the study. This study was carried out in accordance with the recommendations of The University of Bonn Ethics Committee with written informed consent from all subjects. All subjects gave written informed consent in accordance with the Declaration of Helsinki. The protocol was approved by the Ethics Committee of the University of Bonn. None of the subjects reported having any neurological or psychiatric disorders. None of the participants reported having hearing difficulties, wore hearing aids or had cochlear implants. All participants confirmed that they heard the auditory beats clearly at 60 dB sound pressure level. None of the subjects took any medication relevant to the study regularly, or consumed alcohol prior to their participation. One participant reported being a regular smoker.

### Monaural Beat Stimulation

Monaural beats were generated using NCA tone generator software version 2.01 (NCH Swift Sound). We applied beats at frequencies of 6 (theta), 10 (alpha), 40 (gamma) Hz and a control sine wave tone. Beat stimuli frequencies were generated using carrier tones around center frequencies between 110 Hz and 220 Hz. For example, a beat stimulus with a frequency of 6 Hz was generated by applying two carrier tones of 107 Hz and 113 Hz (see also Table 1). A sound pressure level of 60 dB was used for the study. A previous study conducted by our group showed that auditory beat stimulation at 60 dB is sufficient to induce pronounced electrophysiological effects (Becher et al., 2015). To maintain a constant level of attention towards the auditory stimuli during the stimulation period, each beat stimulus increased linearly in pitch over the course of 1 min up to a center frequency of 220 Hz and afterwards decreased again down to 110 Hz, while maintaining an identical beat frequency. Thus, each 5 min stimulation period comprised the pitch sequence increase/decrease/increase/decrease/increase and the same was the case for the 110–220 Hz control tone. Participants were instructed to attend to the reversal in pitch change (from increase to decrease, from decrease to increase) and to press a button on a keyboard at the time point of reversal (the arrow “up” button to indicate a reversal from falling to rising pitch and the arrow “down” button to indicate a reversal from rising to falling pitch). One of two arrows onscreen lit up in response to the decision made by the participants via the button press. The participants received no feedback as to whether their response was correct or not. Beat stimuli were delivered through headphones with closed over-ear head pieces which isolated the participants from external noise (Sennheiser, Wedemark-Wennebostel, Germany) and played through software installed on a laptop computer. Participants wore the headphones throughout the duration of the experimental session and were blinded as to the order of beat stimulation and whether they had listened to the control tone. The overall duration of the beat stimulation was 60 min for each participant for the entire session.

**Table 1:** **A summary of monaural beat stimuli and applied carrier tones**.

Beat frequencies (Hz)	Carrier tones (Hz)
6 (Theta)	107 and 113; increasing to 217 and 223
10 (Alpha)	105 and 115; increasing to 215 and 225
40 (Gamma)	90 and 130; increasing to 200 and 240
110–220 (Control tone)	increasing from 110 to 220

### Experimental Procedure

**Figure 2:**
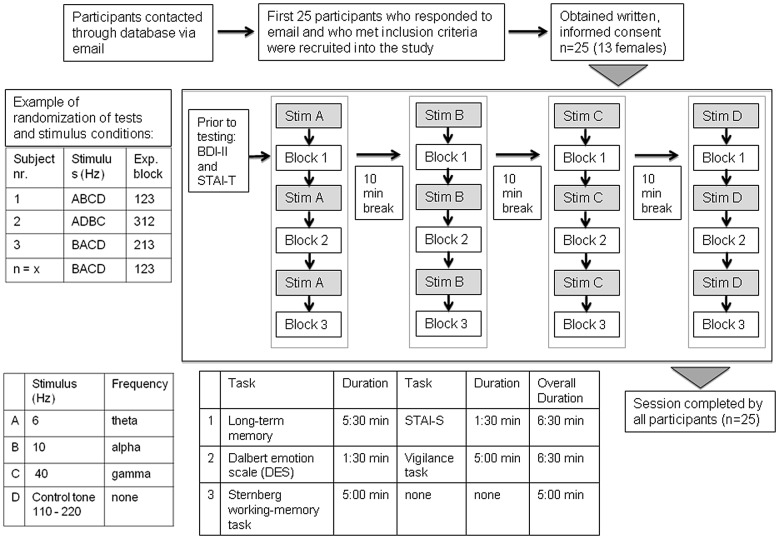
**Overview of participant recruitment and experimental procedure**. After the participants completed the Beck Depression Inventory (BDI) and the State-Trait Anxiety Inventory (STAI)-T, the first beat frequency was presented for 5 min and then once the test block began, the participant was asked to complete each of the questionnaires before the run proceeded to the next beat stimulus. The duration of one session was approximately 3 h.

All experimental sessions were performed in a quiet room and on a laptop computer, where the subjects were told to sit in an upright position with the computer monitor at a comfortable angle. Subjects were informed of the task instructions by watching a short presentation. An overview of the experimental procedure is shown in Figure 2. The test blocks were comprised of a task testing long-term memory and the state anxiety inventory (STAI-S; test block 1), the Dalbert emotion scale and a vigilance task (test block 2) and a variant of the Sternberg working memory task (test block 3). Each test block was then run once for each of the 4 auditory stimulation conditions (beat frequencies: 6 Hz, 10 Hz, 40 Hz and control tone randomized). Before each test block a 5 min stimulation period was applied. The block sequence, as well as the sequence of stimulation conditions was randomly selected for each subject. The test block sequence within each subject was kept the same for the different stimulation conditions. Time taken to complete each test block ranged from 5 min to 7 min. Prior to testing and the onset of beat stimulation, the Beck Depression Inventory (BDI) and the trait anxiety inventory were administered. The cognitive tasks were presented using Presentation software (Neurobehavioral Systems, Berkeley, CA, USA) on a laptop computer and participants were instructed to fill out the rating scales presented on the screen using a standard keyboard. For each stimulation condition, different versions of the working memory and long-term memory tasks were conducted. This means the same tasks were conducted with different stimulus material (i.e., different number sequences for the working memory task and different words for the long-term memory task). The duration of the complete session was approximately 3 h including 10 min breaks between each experimental run.

### Neuropsychological Scales and Cognitive Tasks

#### The State-Trait Anxiety Inventory (STAI)

The STAI inventory was used to evaluate anxiety levels and to differentiate between the participants’ state anxiety (STAI-S) and trait anxiety (STAI-T); i.e., between temporary anxiety related to an event or a situation and anxiety levels that are a personal characteristic (Spielberger et al., 1983). The STAI-S subscore is typically applied to assess anxiety related to different situations, often perceived as dangerous, and refers to how an individual feels at the time of a perceived threat. The STAI-T subscore is associated with feelings of anxiety that an individual experiences on a daily basis. Participants were asked to rate themselves according to the 20 statements contained in the STAI inventory for each subscore with a 4-point Likert scale. The STAI-Trait inventory was completed by each subject prior to the experimental session. The STAI-State inventory was completed during test block 1.

#### Dalbert Emotions Scale (DES)

The DES was used to evaluate mood states (Dalbert, 1992). Participants were asked to rate 19 mood-related statements on a scale from 1 (being least affected) to 7 (most affected). Statements included adjectives describing different emotions underlying mood states. For example, “unhappy” was grouped with “sad” and “sorrowful” to define the mood-state grief. The assessment contained adjectives that equally described negative or positive mood states. From the 19 items assessing emotions, the five mood states grief, hopelessness, tiredness, anger and positive mood can be classified. The DES was derived from the POMS scale (McNair and Heuchert, 2011) and translated into German by Dalbert (1992).

#### Beck Depression Inventory (BDI)

The BDI assesses symptoms of depression by a 21-point multiple-choice questionnaire (Beck et al., 1996). This inventory was completed by each subject prior to the experimental session. The aim of administering the BDI was to evaluate whether possible stimulation-related changes of mood states depend on the presence or absence of symptoms of depression. The BDI was assessed using the following scoring ranges: a score of 0–13: minimal; 14–19: mild; 20–28: moderate; 29–63: severe (Beck et al., 1996).

#### Vigilance Task

For this task participants were asked to attend to six letters (s, d, f, j, k, l) displayed on circular fields on a gray computer screen (similar to the “Wiener-Determinationsgerät”; G. Schuhfried, Schuhfried GmbH). In a random order one of the fields would light up in yellow for 5 s and the subject had to indicate with a press of the corresponding keyboard button that they identified the highlighted field. Subjects were instructed to react as fast as possible and to stay attentive towards the task. Sixty trials were presented and reaction times and button presses were stored in a logfile.

#### Working Memory Task

We applied a serial variant of the Sternberg working memory task with digits (from 1 to 9) as items (Leszczynski et al., 2015). Participants were instructed to maintain one, three, five or seven serially presented digits corresponding to varying loads (loads 1, 3, 5, 7). Each item was presented for 0.5 s, followed by an inter-stimulus interval of 2 s duration. Once the sequence had been presented, participants had to maintain the memory of the entire set for the duration of a retention interval lasting 3 s. Next, a question mark informed participants to type all digits in correct order using a keyboard. Each test version comprised 24 trials with load conditions in random order.

#### Long-Term Memory Task

Subjects were asked to memorize subsequently presented words, which after a distraction task had to be freely recalled (Fernández et al., 1999) and were written down by the experimenter. Words were presented in uppercase letters for 400 ms in a series of 12 words. The interstimulus interval between each word was 2.5 s. Subjects were instructed to use a rote strategy to memorize each word while avoiding memory aids such as making rows, sentences, stories or pictures. During the distraction task subjects were instructed to count backwards in threes, starting at a number between 81 and 99 displayed on the screen. Each test version comprised 24 trials.

### Statistical Analyses

The following dependent variables were evaluated: state-anxiety scores assessed using the STAI-S, scores of the five mood states assessed by the DES, average reaction times and number of correct responses given in the vigilance task, average reaction times and average number of correctly maintained digits in the working memory task, as well as number of correctly recalled words in the long-term memory task. For each domain with more than one dependent variable (DES (5), vigilance (2), working memory (2)) we conducted a one-way repeated-measures MANOVA to evaluate an overall effect of the independent variable BEAT condition (6 Hz, 10 Hz, 40 Hz, control tone) across the dependent measures. Behavioral data from the STAI-S and from the long-term memory task were subjected to one-way repeated measures ANOVAs with the variable BEAT condition. In case of a significant BEAT effect, *post hoc* paired *t*-tests comparing each of the beat stimulation conditions (6 Hz, 10 Hz, 40 Hz) with the control condition were applied (uncorrected for multiple comparisons). For exploratory purposes, we also calculated one-way repeated measures ANOVAs for each of the dependent variables of the domains without significant MANOVA effects. Results of these exploratory ANOVAs have to be regarded with caution. A Shapiro-Wilk test was applied on all data for testing the assumption of normal distribution and Mauchly’s test of sphericity was performed to assess for the assumption of sphericity. *P*-values in the MANOVAs and ANOVAs were Huynh-Feldt-corrected when necessary. Only *p*-values of <0.05 were regarded as statistically significant. Statistical analyses were performed using SPSS 22.0 (SPSS Inc. Chicago, IL, USA).

## Results

### State-Trait Anxiety Inventory (STAI)

**Figure 3:**
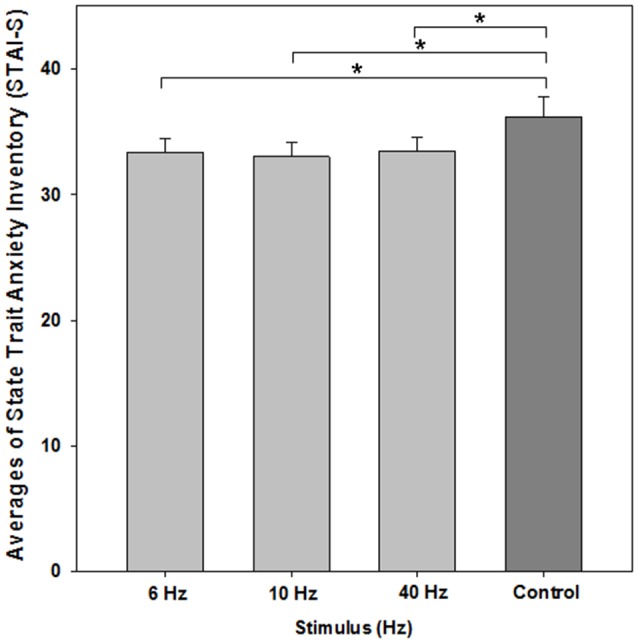
**Average scores of the STAI-S for evaluating state anxiety**. All monaural beat conditions vs. control are shown. A reduction in anxiety scores was statistically significant for each beat condition. Asterisks indicate significant differences between beat stimulation and control condition (paired *t*-tests, *p* < 0.05). Error bars indicate standard error of mean (SEM).

The one-way repeated measures ANOVA showed a significant effect of BEAT condition on state-anxiety compared to the control condition (*F*_(3,72)_ = 4.669; *p* = 0.012; *ɛ* = 0.719). *Post hoc* paired *t*-tests revealed that each beat condition differed from control (6 Hz: *M* = 33.36, SEM 1.133, *t*_(24)_ = −2.395, *p* = 0.025; 10 Hz: *M* = 33.04, SEM = 1.09, *t*_(24)_ = −2.675, *p* = 0.013; 40 Hz: *M* = 33.40, SEM = 1.06, *t*_(24)_ = −2.355, *p* = 0.027; control condition: *M* = 36.20, SEM = 1.571; see Figure 3). There was no significant effect between beat conditions (each *p* > 0.55).

To further evaluate whether the changes in state-anxiety depend on the level of trait-anxiety, we additionally performed a two-way ANOVA for the variable state-anxiety with the within-subject factor BEAT, and the between-subject factor TRAIT-ANXIETY (median split: low vs. high values). This ANOVA revealed no interaction (*F*_(3,69)_ = 1.373; *p* = 0.263; *ɛ* = 0.704). We observed a trend for a main effect of TRAIT-ANXIETY levels on state anxiety (*F*_(1,23)_ = 2.948, *p* = 0.099).

In order to investigate whether the sequence of beat stimulation conditions impacts on any observed effects for state-anxiety, we additionally conducted two-way ANOVAs with BEAT condition (6 Hz vs. control; 10 Hz vs. control; 40 Hz vs. control) as repeated measure and POSITION of the beat stimulation condition (A, B, C, D; see Figure 2) as independent variable. This means, for each stimulation condition (e.g., for 6 Hz) such an ANOVA was calculated, where the variable POSITION indicated the position of this stimulation condition (e.g., 6 Hz) in each subject. These ANOVAs revealed no interactions between the factors BEAT condition and POSITION (6 Hz vs. control: *F*_(3,21)_ = 1.084, *p* = 0.378; 10 Hz vs. control: *F*_(3,21)_ = 0.080, *p* = 0.970; 40 Hz vs. control: *F*_(3,21)_ = 1.703, *p* = 0.197).

### Dalbert Emotion Scale (DES)

An overall MANOVA revealed no significant effect of BEAT stimulation on the DES measures (Wilk’s lambda = 0.789; *F*_(15,188)_ = 1.123, *p* = 0.34). The one-way repeated measures ANOVAs showed a significant effect of BEAT stimulation on the “grief” item of the DES, as defined by the adjectives “unhappiness, sadness and sorrow” (*F*_(3,72)_ = 2.754; *p* = 0.049; *ɛ* = 0.993). A significant reduction of the “grief” score was observed for the 40 Hz monaural beat condition (*M* = 3.40; SEM = 0.200; *t*_(24)_ = −2.268; *p* = 0.033), as well as a trend for the 10 Hz beat condition (*M* = 3.52, SEM = 0.217; *t*_(24)_ = −1.768; *p* = 0.09), when compared to the control condition (*M* = 4.00; SEM = 0.351). There was no difference for the 6 Hz beat condition (*M* = 3.92; SEM = 0.326; *t*_(24)_ = −0.449; *p* = 0.770) vs. control condition. As multiple measures were tested, and the MANOVA revealed no significant effect of BEAT stimulation, this result has to be regarded with caution.

To further evaluate whether the changes in grief scores depend on the presence or absence of symptoms of depression, we additionally performed a two-way ANOVA for the variable “grief” of the Dalbert emotion scale with the within-subject factor BEAT and the between-subject factor BDI level (median split: low vs. high BDI values). There was no main effect of the BDI level (*F*_(1,23)_ = 1.505; *p* = 0.232) and no significant interaction (*F*_(3,72)_ = 1.755; *p* = 0.164; *ɛ* = 1.00).

The ANOVAs for the items hopelessness (*F*_(3,72)_ = 0.169; *p* = 0.881; *ɛ* = 0.802), tiredness (*F*_(3,72)_ = 0.755; *p* = 0.581; *ɛ* = 0.957), anger (*F*_(3,72)_ = 2.283; *p* = 0.124; *ɛ* = 0.551) and positive mood (*F*_(3,72)_ = 0.454; *p* = 0.709; *ɛ* = 0.965) did not show significant effects.

#### Vigilance Task

An overall MANOVA revealed no significant effect of BEAT stimulation on the vigilance measures (Wilk’s lambda = 0.872; *F*_(6,142)_ = 1.675, *p* = 0.13). Moreover, no significant ANOVA effects of BEAT stimulation were observed on the number of correct responses (*F*_(3,72)_ = 1.643; *p* = 0.190; *ɛ* = 0.950) or on reaction times (*F*_(3,72)_ = 1.816; *p* = 0.179; *ɛ* = 0.581). The average performance scores (mean and SEM) for the different beat conditions are listed in the following. Percentage of correct responses: 6 Hz (*M* = 98.3%; SEM = 0.33%); 10 Hz (*M* = 97.7%; SEM = 0.36%); 40 Hz (*M* = 97.3%; SEM = 0.52%); control (*M* = 98.0%; SEM = 0.48%). Reaction times: 6 Hz (*M* = 560.5 ms; SEM = 12.6 ms); 10 Hz (*M* = 580.1 ms; SEM = 16.7 ms); 40 Hz (*M* = 597.2 ms; SEM = 25.5 ms); control (*M* = 576.6 ms; SEM = 13.8 ms).

#### Working Memory Task

An overall MANOVA revealed no significant effect of BEAT stimulation on the working memory measures (Wilk’s lambda = 0.933; *F*_(6,142)_ = 0.835, *p* = 0.55). Moreover, repeated measures ANOVAs revealed no significant effect of BEAT stimulation on working memory performance in terms of the number of correctly maintained digits (*F*_(3,72)_ = 0.648; *p* = 0.587; *ɛ* = 1.00) or on reaction times (*F*_(3,72)_ = 1.419; *p* = 0.244; *ɛ* = 1.00). The average performance scores (mean and SEM) for the different beat conditions are listed in the following. Percentage of correctly maintained digits: 6 Hz (*M* = 79.5%; SEM = 2.4%); 10 Hz (*M* = 78.9%; SEM = 2.8%); 40 Hz (*M* = 77.6%; SEM = 2.4%); control (*M* = 80.8%; SEM = 1.9%). Reaction times: 6 Hz (*M* = 1135.7 ms; SEM = 54.2 ms); 10 Hz (*M* = 1186.8 ms; SEM = 53.8 ms); 40 Hz (*M* = 1184.9 ms; SEM = 36.2 ms); control (*M* = 1104.7 ms; SEM = 56.0 ms).

#### Long-Term Memory Task

Repeated measures ANOVA showed no significant effect of monaural beat stimulation on long-term memory performance in terms of correctly recalled words (*F*_(3,72)_ = 0.963; *p* = 0.410; *ɛ* = 0.918). The average performance scores (mean and SEM) for the percentage of correctly recalled words are: 6 Hz (*M* = 55.8%; SEM = 2.7%); 10 Hz (*M* = 58.3%; SEM = 3.0%); 40 Hz (*M* = 61.8%; SEM = 2.9%); control (*M* = 58.8%; SEM = 3.1%).

## Discussion

In the present study we investigated the impact of a short duration monaural beat stimulation on measures assessing state anxiety levels and mood states in addition to two types of memory tasks (long-term and working memory) and a vigilance task. We report that monaural beat stimulation at gamma (40 Hz), theta (6 Hz) and alpha (10 Hz) frequencies was effective in reducing state-anxiety scores (as assessed using the STAI-S). Moreover, an exploratory result of our study is that gamma and alpha stimulation reduced ratings for the item grief in the DES, compared to the control condition. However, this result has to be regarded with caution as a MANOVA was non-significant across the five DES items. No significant effects were found on long-term memory (number of correctly recalled words), working memory (correctly maintained digits and reactions times in a variant of the Sternberg task) or vigilance levels (correct button presses and response latencies). There was also no interaction between the beat-related reduction in grief scores and the level of symptoms of depression in the BDI, and no interaction between beat-related reduction of state anxiety and the level of trait anxiety. Since we investigated a healthy population, the spread in BDI (mean score 4.92 ± SD: 4.3; scores can range between 0 and 63, with higher scores indicating greater depression levels) and trait-anxiety levels (mean score 36 ± SD: 8.3; scores can range between 20 and 80 with higher scores indicating greater anxiety levels) may have been too small to detect such interactions.

Findings from our current investigation using monaural beats are in line with earlier studies using binaural beats, reporting that beat stimulation modulates levels of anxiety and enhance feelings of well-being (Le Scouarnec et al., 2001; Padmanabhan et al., 2005; Wahbeh et al., 2007a; Weiland et al., 2011). In the study by Weiland et al. (2011), reductions of state-anxiety in a binaural beat stimulation group compared to ambient noise stimulation and no sound/headphones only control groups was reported with effect sizes (Cohen’s d; Cohen, 1988) of 0.35 and 0.49 based on the data provided in the article. In the beat stimulation group the reduction of state-anxiety post- vs. pre-intervention had an effect size of 0.52. In the study by Wahbeh et al. (2007a) a reduction of trait-anxiety after vs. before a 60-day binaural beat intervention was reported with an effect size of 0.83. The data provided in Le Scouarnec et al. (2001) and Padmanabhan et al. (2005) doesn’t allow for the calculation of effect sizes. For the effects of monaural beat stimulation on state-anxiety we observed in the current study effect sizes of 0.58 (6 Hz vs. control), 0.61 (10 Hz vs. control) and 0.39 (40 Hz vs. control), which are in a similar range as the effects reported in the studies on binaural beat stimulation. However, we did not directly compare the effects of binaural vs. monaural beats on anxiety levels in this study in order not to overburden the study design, and our participants. It is of interest to note that although there is evidence that cortical responses to monaural beat stimulation are more prominent than those to binaural beats (Schwarz and Taylor, 2005; Pratt et al., 2010), the majority of studies using auditory beat stimulation have been performed using binaural beats as stimuli (see Chaieb et al., 2015 for a review). Here we aimed primarily to examine whether monaural beats would have a similar impact on state-anxiety, as that of binaural beat stimulation.

Previous research suggests that binaural beats reduce anxiety levels (Padmanabhan et al., 2005; Wahbeh et al., 2007a; Weiland et al., 2011) and may possibly modify aspects of mood and cognition—including memory (Wahbeh et al., 2007b; Ortiz et al., 2008), attentional processes (Kennel et al., 2010), and vigilance (Lane et al., 1998). Padmanabhan et al. (2005) treated patients suffering from pre-operative anxiety using binaural beats in the delta frequency range. Patients listened to either a 10 min recording of binaural beats or a sham-like audio sequence. The authors reported a 26.3% decline in state-anxiety scores in the binaural beat audio group, compared to a 11.1% decline in the placebo audio group (Padmanabhan et al., 2005). In a pilot study, daily applications of binaural beats in the delta/theta frequency range for 30 min was used to treat patients suffering from mild anxiety disorders (Le Scouarnec et al., 2001). Patients listened to the beat files across a period of 1 month, and indicated anxiety scores pre- and post-stimulation using the STAI. An overall reduction in state-anxiety ratings, as well as an increase in the number of times patients chose to listen to the beat recordings was reported. A similar study on patients suffering from anxiety conducted in an emergency department reported a significant decline in state-anxiety scores by 10%–15% compared to the control conditions (ambient noise and no sound/headphones only) in which binaural beats were absent (Weiland et al., 2011). Stimuli were binaural beat frequencies of 10 Hz embedded into natural sounds. The intervention was applied for 20 min and patients were requested to complete the STAI in order to assess anxiety scores. Binaural beats in the beta (16 and 24 Hz) and theta/delta range (1.5 and 4 Hz) were also reported to be associated with less negative mood states (Lane et al., 1998).

Accordingly, anxiety and mood-related disorders may offer a potential target for auditory beat stimulation applications. Previous studies addressing the electrophysiological correlates of anxiety-related disorders such as generalized anxiety disorder, post-traumatic stress disorder (Rabe et al., 2006) and obsessive compulsive disorder (Insel et al., 1983) often revealed increases of baseline delta and theta activity (i.e., physiological deactivation) in patients vs. healthy controls, while findings for higher frequency activity were rather divergent (for an overview see Clark et al., 2009). Moreover, several studies suggested that the topography of frontal alpha activity in patients suffering from anxiety and mood disorders differs from healthy controls (e.g., Smit et al., 2007; Mathersul et al., 2008) and generally may reflect psychological well-being (e.g., Tomarken et al., 1992; Urry et al., 2004). A recent intracranial EEG study from our group demonstrated enhancements of gamma power due to stimulation with monaural 40 Hz beats, decreases of theta power due to stimulation with monaural 5 Hz beats, and enhancements of alpha phase synchronization related to application of monaural 10 Hz beats (Becher et al., 2015). Based on these data one may tentatively speculate that the behavioral effects reported in the present study for monaural 6 Hz and 40 Hz beats are related to a reduction of elevated baseline theta activity or a counterbalancing by an enhancement of gamma activity (i.e., physiological activation). Furthermore, the anxiety-reducing effect found for monaural 10 Hz beats may be related to changes in frontal EEG synchronization, since altered frontal alpha lateralization has been reported to reflect anxiety disorders (e.g., Mathersul et al., 2008). However, further in-depth investigations are needed to clarify the electrophysiological mechanisms underlying the observed behavioral effects.

To summarize, we show that a short application of monaural beat stimulation in healthy human participants is able to reduce feelings of anxiety. Although auditory beat research has tended toward using binaural beats as stimuli, our findings suggest a potential role for monaural beat stimulation in treating symptoms of anxiety disorders, in a fast and non-invasive manner. Future studies will aim at identifying ideal target populations for monaural beat stimulation and at optimizing stimulation parameters, for instance, by increasing the stimulation duration or by repetitive applications of the beat stimuli.

## Author Contributions

ECW, CH, NA and JF generated the concept of the study and designed the experimental protocol. ECW performed the data collection. ECW, LC and JF performed the data analysis. LC, ECW, CH, NA and JF wrote the manuscript.

## Conflict of Interest Statement

The authors declare that the research was conducted in the absence of any commercial or financial relationships that could be construed as a potential conflict of interest.
